# Embodiment and Estrangement: Results from a First-in-Human “Intelligent BCI” Trial

**DOI:** 10.1007/s11948-017-0001-5

**Published:** 2017-11-11

**Authors:** F. Gilbert, M. Cook, T. O’Brien, J. Illes

**Affiliations:** 1 Centre for Sensorimotor Neural Engineering, Department of Philosophy, University of Washington, Seattle, WA USA; 2 National Core for Neuroethics, Division of Neurology, Department of Medicine, University of British Columbia, Vancouver, BC USA; 3 Australian Research Council DECRA Fellow, University of Tasmania, Hobart, Australia; 4 Department of Medicine, The Royal Melbourne Hospital, University of Melbourne, Parkville, Australia; 5 Department of Neurology, St. Vincent’s Hospital, Melbourne, Australia

**Keywords:** Advisory system, Agency, Artificial Intelligence, Assistive system, Autonomy, Brain computer interfaces, Brain machine interfaces, Brain implant, brain device, capacities, Control, Embodiment, Estrangement, Identity, Implantable device, Predictive device, Phenomenology, Predictive brain devices, Qualitative interviews, Self

## Abstract

While new generations of implantable brain computer interface (BCI) devices are being developed, evidence in the literature about their impact on the patient experience is lagging. In this article, we address this knowledge gap by analysing data from the first-in-human clinical trial to study patients with implanted BCI advisory devices. We explored perceptions of self-change across six patients who volunteered to be implanted with artificially intelligent BCI devices. We used qualitative methodological tools grounded in phenomenology to conduct in-depth, semi-structured interviews. Results show that, on the one hand, BCIs can positively increase a sense of the self and control; on the other hand, they can induce radical distress, feelings of loss of control, and a rupture of patient identity. We conclude by offering suggestions for the proactive creation of preparedness protocols specific to intelligent—predictive and advisory—BCI technologies essential to prevent potential iatrogenic harms.

## Introduction

As implantable Brain Computer Interface (BCI) research moves rapidly ahead, concern about its potential effects on patients’ sense of self, autonomy and identity is growing (Klein 2015; Glannon 2016; Klein and Jeffrey 2016; Clausen 2013; Gilbert 2015a, b; Brown et al. 2016; Hildt 2015).^1^ For instance, the evolution of prosthetic limbs controlled through BCI introduces questions about body ownership, self-image and self-understanding. To what extent does an implantable BCI device become incorporated into a patient’s sense of self? To what degree does an implantable BCI device intrude on the patient’s individual capacities and lead to iatrogenic harms? BCI innovations may blur the boundaries between a patient’s sense of identity and computerised implantable devices in unprecedented ways. BCI technologies offer great control at the level of neural circuits, but the extent to which this grasp on neuronal function affects the patient’s sense of control at the psychological level is still uncharted territory (Glannon and Ineichen 2016).

Questions today about potential BCI-induced effects on patients are directly imported from clinical data compiled from reports and studies of open-loop, historically longer standing technologies such as deep brain stimulation (DBS). It is unclear, however, how DBS studies translate to closed-loop BCI research (Kellmeyer et al. 2016). With the revolution in BCI-based neural engineering on the horizon, there is a pressing need to explore and address the potential effects of BCIs as they may impinge on concepts of self, control and identity.

This article addresses the knowledge gap by providing data obtained from a first-in-human clinical trial involving six patients implanted with a new generation of predictive and advisory BCI implants (Cook et al. 2013). These artificially intelligent—predictive and advisory—implants detect specific neuronal activity patterns believed to be precursors to neuronal events, and then provide information in the form of visual or auditory signals to help patients initiate preventative/therapeutic responses. The first in-human trial on which this study is based, focused on epilepsy, a neurological condition that affects more than 60 million people worldwide. 30–40% of these cases are not adequately controlled with current treatments (Kwan and Brodie 2000). By preparing the implanted patient to anticipate a seizure, the technology allows the patient to take precautionary steps such as taking medication or getting situated in a safe place (Cook et al. 2013).

## Methods

We explored perceptions of self-change as provided by six patients who volunteered to be implanted with the first-in-human, experimental BCI advisory brain devices capable of predicting epileptic seizures and to participate in our study. The trial was conducted to provide safety and proof-of-concept efficacy data for BCI seizure detection in patients with medically refractory epilepsy. The BCI was capable of learning brain activity patterns from individual based on artificial intelligence techniques (Gardner et al. 2006). A patient-designed algorithm was created following data collection phases, during which intracranial electroencephalograms (EEG) were performed for assessments of individual epilepsy-monitoring (Cook et al. 2013). The BCI device was composed of two silicon implantable lead assemblies containing eight contacts collecting intracranial EEG on patient cortical surface (see image). The leads were connected, through the neck, to a subclavicularly placed implantable telemetry unit, which was inductively recharged through an external charging accessory (see image) (Cook et al. 2013). Wirelessly transmitted predictive data were displayed on a hand-held personal advisory device (see image). The trial was conducted across three clinical centres in Australia—Austin Health, The Royal Melbourne Hospital, and St Vincent’s Hospital (Cook et al. 2013). The BCI devices were implanted in fifteen patients overall.

We used qualitative methodological tools grounded in phenomenology to conduct in-depth, open-ended, semi-structured individual interviews with each of the six patients who were drawn from three Australian states, namely Victoria, New South Wales and Tasmania.^2^ All interviews were conducted in English. Interviews were based on an adapted version of the qualitative instrument first developed and tested in Gilbert (2015a, b) and further elaborated in Gilbert et al. (2017) and Gilbert et al. (In Press) (“Appendix”). Interviews were transcribed verbatim. Contents were then manually coded and finally analysed by regrouping patients’ subjective experiences into common themes and clusters.

The qualitative approach allowed us to capture unique, first-person perspectives that are not identifiable with standardized questionnaires and scales. By using phenomenological methods, we were able to increase opportunities for finding fundamental, patient-specific narratives and perceptions based on lived experiences (Gallagher 2003). The main findings as defined by their prominence in the narratives are highlighted and summarized in excerpts.
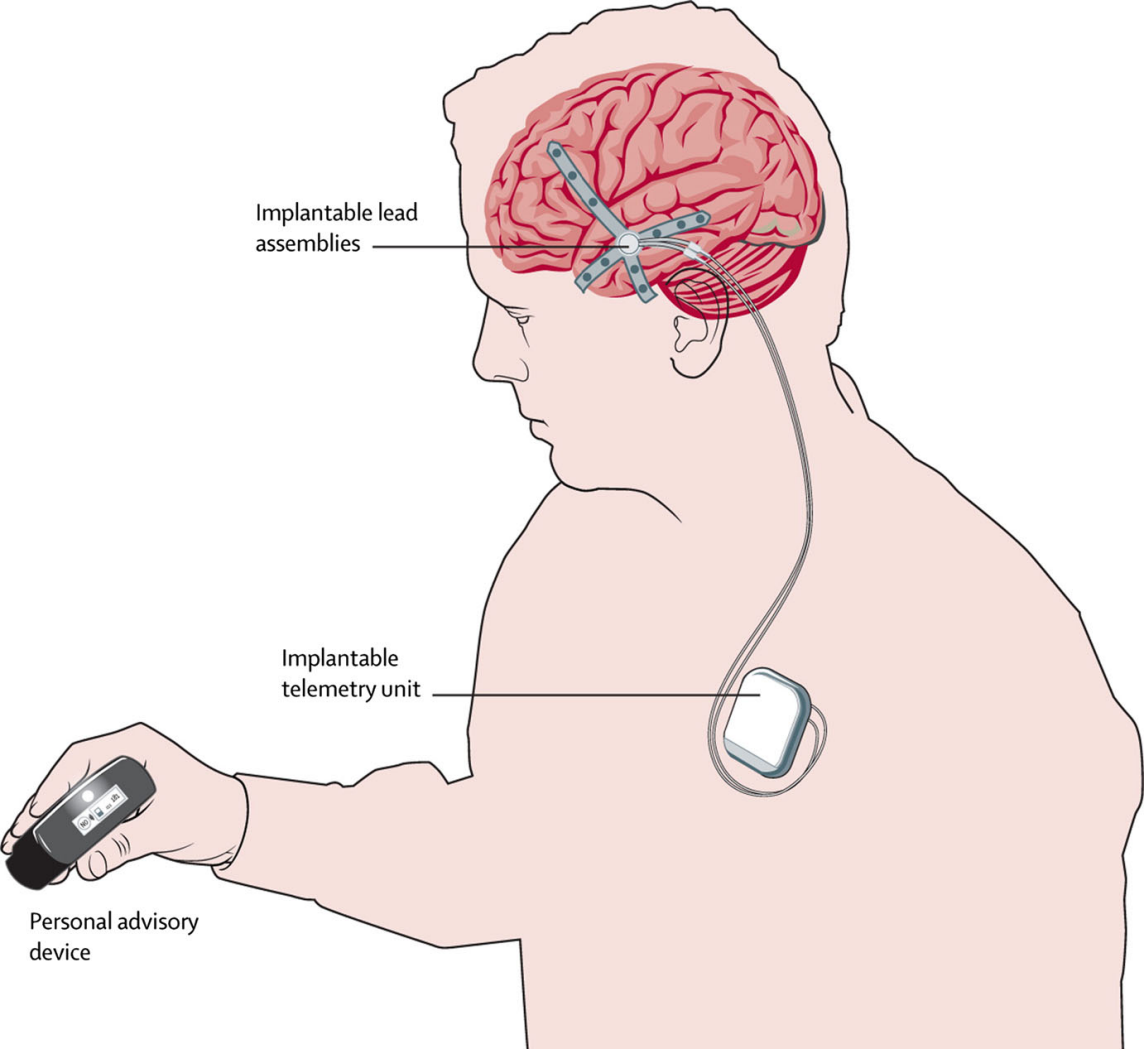


Copyright^©^ 2013 Elsevier Ltd.

Reprinted from (Cook O'Brien Berkovic et al. 2013) Prediction of seizure likelihood with a long-term, implanted seizure advisory system in patients with drug-resistant epilepsy: A first-in-man study. The Lancet Neurology. 12(6): 563–571 with permission from Elsevier.

## Results

We collected a total of six interviews: 6.25 h of in-person interview data with a mean duration of 1:05 h from five patients, and one interview by email-correspondence.

Three major phenomenological clusters emerged from our analysis: (a) subjective experience of living with chronic epilepsy; (b) subjective perception of BCI-induced sense of control; and (c) subjective feeling of BCI-effects on sense of self. These clusters were populated by patients’ key answers and quotes (Table 1). As this was a qualitative study based on first-person narratives, the main results are highlighted and given in excerpts displayed in Table 1.

**Table 1:** Excerpts from patients’ reported subjective experiences of using BCIs

Patient	(a) Experience with chronic epilepsy	(b) BCI-induced sense of control and empowerment	(c) BCI effects on sense of the self
Patient 1	“Seizures interrupt my life”.	“I felt more in control when I used the device. I could push on and do what I wanted to do”.	“I don’t think it changed me as a person, but it gave me more confidence and control”.
Patient 2	“I’ve got to say that [epilepsy]’s part of me”.	“[The BCI] gave me more confidence to do things that I wouldn’t necessarily and normally do”.	“[The effects are] a natural consequence of the development of the algorithm”.
Patient 3	“I just kind of pretended that [epilepsy] didn’t really exist, I didn’t see myself as an epileptic”.	“[The BCI] made me feel I had no control. So I didn’t have control over what I was going to do”.	“[The BCI] made me feel that I was always differents [from] everyone not just in the moment of the seizure […] I got really depressed”.
Patient 4	“It’s been part of my life now, it’s part of [me] now”.	“I just ignore [the BCI] anyway I just kept on doing my own thing”.	“I wouldn’t say the technology was an integral part of me”.
Patient 5	“[Epilepsy] is part of me”.	“[The BCI] gave me more confidence in some respects”.	*Patient 5 did not report any experiences about his self.*
Patient 6	“I see my epilepsy-I’ve never liked it-it’s been an opposition to me and it’s caused me a lot of depression, anguish and a lot of teasing”.	“[The BCI] changed my confidence, it changed my abilities”.	“[The BCI] was me, it became me, […] with this device I found myself”.

When looking more carefully at phenomena reported by patients (Table 1), especially those concerning how BCIs induced a sense of empowerment (Cluster B), we observed that four patients experienced, in different degrees, an increased sense of control, leading to a sense of substantial augmented confidence. Patients reported that this confidence enabled them to act unimpeded by the constant risk of otherwise unpredictable seizures. In this regard, the degree of control experienced appears to be directly proportional to the degree of confidence provided by BCI implants. For instance, Patient 1 reported: “I felt more in control when I used the device. I could push on and do what I wanted to do”, alluding to his new capacity to undertake activities promoting his autonomy (e.g., playing cricket). This is further echoed by Patient 5 who explained: “[The BCI] gave me more confidence in some respects”. Patient 6 also self reported: “With the device I felt like I could do anything—I can do this—I can do everything I want to do”. In both cases, patients explored novel degrees of freedom of decision to accomplish new activities, as epilepsy was obstructing their independence and autonomy to make decisions and act in specific ways. Patient 2 claimed: “[The BCI] gave me more confidence to do things that I wouldn’t necessarily and normally do”. Patient 6 also stated: “[the BCI] changed my confidence, it changed my abilities”.

**Fig. 1:**
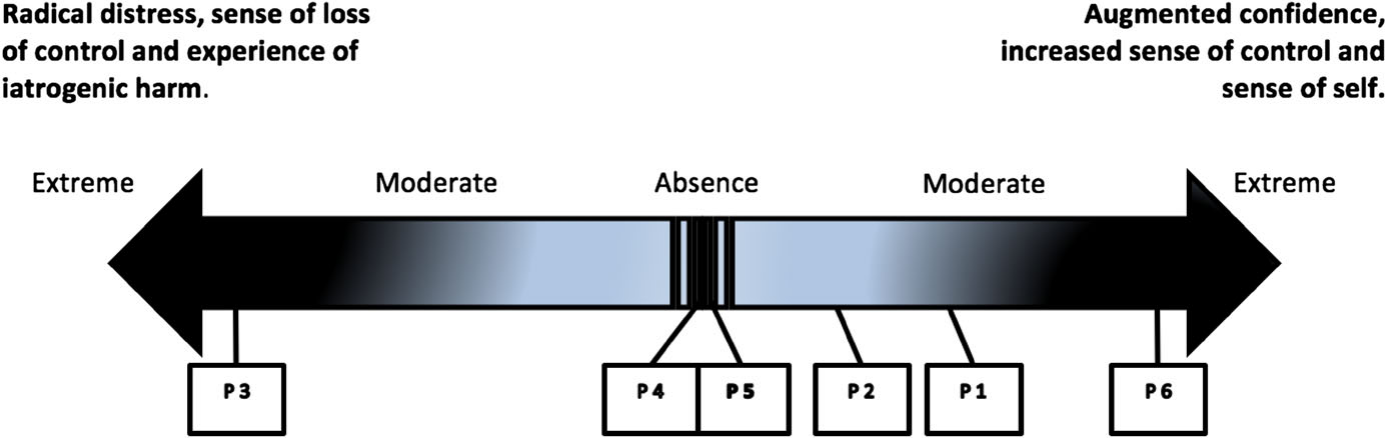
Patients’ perceptions on a continuum from radical distress, sense of loss of control and experience of iatrogenic harm, to augmented confidence, increased sense of control and sense of self

However, two patients did not corroborate the findings related to a post-implantation augmented and amplified sense of control and confidence. Patient 3 reported “[the BCI] made me feel I had no control. So I didn’t have control over what I was going to do”. Patient 4 declared “I just ignored [the BCI signals] anyway. […] I just kept on doing my own thing”. Figure 1 illustrates the range of subjective experiences reported by the six patients interviewed.

The findings also suggest that BCI devices intruded on patients’ postoperative sense of self. Patient 3 and Patient 6 reported experiences located at opposite ends of the continuum seen in Fig. 1. Patient 6, who perceived that her disease caused her a “lot of harm and anguish”, reported how the implanted BCI device forecasts were naturally integrated into her existence and became an indispensable embodied component of her self:

Patient 6: “[The device] was like an alien at first, […] you grow gradually into it and get used to it, so it then becomes a part of everyday, it’s there every day, it’s there every night you go to bed and you put it in a place that it can still read you so it’s like a teddy bear. Really it’s there and you can see it, you know that if you open your eyes so it’s always there, it follows you through the shower everywhere and it becomes part of you. Because that’s what it did, it was me, it became me, […] with this device I found myself”.

Describing her experience further, she added:

Patient 6: “It changed who that person was then and I found myself changing… growing I suppose and it changed my confidence, it changed my abilities – it changed how stressed I was, how well I slept, then I could make decisions without having to worry about what might or might not happen. […] With the device I felt like I could do anything – I can do everything I want to do I was more capable of making good decisions – not bad decisions – because there’s been times where I’ve made bad decisions […] I can bake safely, I can bath shower safely. So it gave me a new lease on life and nothing could stop me”.

Patient 3 reported contrasting experiences on the continuum^3^:

Patient 3: “I had [epileptic seizures] since I was sixteen. I pretended that they didn’t really exist for a while. And then I was fine but I just kind of pretended that it didn’t really exist. I didn’t really see myself as an epilectic. […] [The BCI] made me feel like I was just sick all the time. […] It also made me feel like I was different to everyone else […] I didn’t want to show anyone that I have it and it was like – I only have epilepsy. […] I felt so weird all the time; before it, I only felt strange having the seizure”.

The experience of being implanted with a BCI advisory system drastically intruded on the sense of self of Patient 3, making her feel constantly confronted:

Patient 3: “because it was always beeping and always red, it made me feel like *I had no control*. So *I didn’t have control over what I was going to do*.[…] I got really depressed. […] I had been depressed before, I’d been very depressed, and I knew what depression was, …I knew what depression felt [like]; ‘know I’m getting depression’, I remember how depression felt, I remember very much what depression felt and I could feel very much exactly the same”.

## Discussion

The experience of a radical life transformation from living with the constant uncertainty of not knowing if and when a next adverse neurologic event will come, to successfully managing and preventing it with the assistance of an implantable BCI device, might first appear as a boost for patient autonomy. However, this study with the first six patients ever tested with an intelligent BCI for epilepsy suggests that the transformation does not necessarily bring with it only a sense of enhanced control and continuity in daily activities, but may have the effect of self-estrangement. Even though the number of patients enrolled in this trial is small, and the data are qualitative and not generalizable to a larger population, the findings are of important ethical concern and could serve to inform future clinical applications of such devices and the research that leads up to them.

We found in our sample that intelligent BCIs were perceived as beneficial by most patients in terms of an associated increased sense of control over their daily activities (Patients 1, 2, 5, 6). For most patients, it seems these intelligent BCI predictive functionalities generated changes in the perceived degree of control over their daily life activities, rather than changes in what constituted them as a person. However, one patient testified to strong experiences of embodied fusion of self with the device resulting in feelings of a new postoperative identity (Patient 6). Importantly, we found one case (Patient 3) of a patient experiencing distress and feeling a loss of control.

The narratives of Patient 6, for example, refer to a discontinuous transition from who she was pre-implantation to who she became after the intervention: the device helped the patient find what she understood to be her ‘true self’. In her case, she not only became functionally dependent on predictions of the BCI advisory system, but the technology was incorporated into her sense of self: “it becomes part of you. Because that’s what it did, it was me, it became me, […] with this device I found myself”. Therefore, for Patient 6, the BCI device became an extension of her self and fused with part of her body: i.e., an integral component of her embodied personal identity. As such, it affected her aspirational goals and augmented her activities of daily living as part of the process of self-definition and self-expression.

The experiences reported by the patients also suggest that the BCI advisory devices can induce an abrupt transition in a patient’s self-understanding as associated with the disappearance *absence* of symptoms. Finding onself changed through implantation can also result in a sense of postoperative self-estrangement. It is important to note that postoperative self-estrangement should be distinguished by deteriorative and restorative features (Gilbert et al. 2017; Gilbert 2017). The notion of self-estrangement exists in association with certain common feelings of a (1) loss of control (Patient 3 “[the BCI] made me feel I had no control” and (2) distorted perceptions of one’s capacities (Patient 6: “With the device I felt like I could do anything […] nothing could stop me”)—the first (1) being mostly associated with a deteriorative sense of the self, and the second (2) largely related to a restorative sense of the self.^4^ It should be recognized that drawing on subjective or first-person phenomenological accounts of how a person feels about or experiences the BCI implant does not provide us with enough context to assess the objective accuracy of these accounts. Self-estrangement may occur, but is not necessarily perceived by the implanted patient (Thomson and Segrave 2017).^5^

With respect to restorative features of self-estrangement, some patients perceived them as naturally occurring (Patient 2: “It is a natural consequent of the development of the algorithm”). The explanation for this may reside in the experience of changed embodiment with the patient coming to feel that the device is authentically a part of themselves. In this study, patients’ self-reports indicate that implanted advisory devices are experienced as increasing restorative powers (i.e., empowerment) over the self (Patient 6: “[The device] changed who that person was then and I found myself changing”).

On the other hand, in terms of deteriorative features of estrangement, Patient 3 experienced feeling a loss of control; that reflect an involuntary shift in their experience of the self. Some feelings of postoperative deterioration of the self were associated with a sense of powerlessness, resulting in severe distress. The notion of postoperative powerlessness has been associated with an inaptitude to overcome some induced psychological realities, for instance Patient 3 seemed not able to regain control of her depressive feelings. From this perspective, the prolonged absence of the capacities to regain control may have contributed to turning estrangement into harm (Gilbert 2015a, b). This finding supports the hypothesis that postoperative feelings of powerlessness play a crucial role in causing iatrogenic harm (Gilbert 2013a, 2015b).

Patient 3 presents an illustration of how the experience of having a BCI constantly monitor, identify and translate one’s brain activities impacts the implanted patient’s self-understanding. Sudden exposure to medical information that predicts upcoming chronic symptoms resulted in dramatic negative consequences to the self-image of certain patients. In the case of Patient 3, the information disclosed and exposed by the device did not corroborate the patient’s self-image and self-narrative. Patient 3 never associated herself with epilepsy: “I just kind of pretended that it didn’t really exist. I didn’t really see myself as an epilectic”.

However, being constantly alerted by the machine impressed upon her the reality of her symptoms, thus providing corroborating evidence of her disease state. This process exacerbated her predisposition to depression; in effect, she was unable to manage the information load returned by the device. In turn, this provoked a dramatic clash with her self-understanding: “I felt so weird all the time, before it I only felt strange having the seizure” (Patient 03).

## Ethical Recommendations

The findings provide evidence useful in elaborating ethical guidance concerning clinical protocols of BCIs. Given the current limited amount of knowledge about the impact of BCIs on the implanted patient’s sense of identity, a first recommendation is to encourage further research. This research should assess whether the restorative or deteriorative effects of BCIs are a temporary phenomenon or whether they will last for months or years. Even if some deteriorative effects progressively disappear after a few weeks, it is still ethically imperative that patients are informed about the transient character of the potential phenomenon.

In view of the findings, especially the case of Patient 3, our second recommendation is that it may be permissible to decline implanting BCIs in patients who experience preoperative self-image issues in relation to their pathology. This recommendation should hold until better knowledge is gained from the potential deteriorative effects of BCIs on patients. Cases where implanted devices have a radical impact on patients’ sense of self may be significantly correlated with key identity development periods. Wilson et al. (2001a, b, 2007, 2010) have observed that greater changes in self-image are associated with seizure onset during, or before, adolescence, and lesser changes in self-image were linked with adult onset. One explanation for the latter claim is that it may be the case that by the time adulthood is reached, the relevant patients will have an already established sense of the self that, as such, is more impervious to being affected (Wilson et al. 2001a, b, 2007, 2010). In this regard, the findings in the study suggest that Patient 3 did not perceive herself as an epileptic in early adulthood, despite experiencing seizure symptoms. In that respect, the BCI disclosed information that did not corroborate Patient 3’s self-image and self-narrative, which align with Wilson and colleagues’ conclusion. In parallel, in another study conducted by one of us with 17 patients implanted with DBS devices, it was observed that the more patients preoperatively felt alienated by their illness, the more they experienced postoperative self-estrangement (Gilbert et al. 2017). These findings are also further evidence that self-concept and self-image prior to implantation are related to potential postoperative impact on patient sense of self and identity.

Taking time to prepare patients for potential radical changes in self-image following implantation is essential. A lack of preparedness to deal with restorative or deteriorative outcomes could make patients and their families more vulnerable to negative outcomes and lead to distress. Clinical preparation should focus on introducing regulatory preoperative psychometric self-image measurements to identify links between patient self-image and illness. These psychometric self-image measures could prepare patients in anticipating radical self-change due to abrupt deteriorative or restorative effects of BCIs. Limiting access to BCI experimental trials for some patients experiencing preoperative self-image issues in relation to their illness is a precautionary step. Given the current state of the art, BCI experimental trials are not treatment; they are not a therapeutic intervention within a consented study protocol. If some patients with self-image issues were to be excluded from trials this would not deny them access to potential treatment. Recommending exclusion of some patients from participating in experimental trials would help to prevent relevant harms within the given experimental regimen.

## Conclusion

This study provides new knowledge about the impact of novel artificially intelligent BCI technologies on end-users. Intelligent closed-loop BCI devices can have a radical and profound influence on patients’ postoperative sense of control and self. However, it is still unclear how the potential effects on embodiment or the experience of self-estrangement are caused. At this time it is relatively difficult to isolate the cause of these potential postoperative changes, though they have been associated with BCI implants in our study. To the best of our knowledge, no study should claim that potential postoperative personality changes can be predicted solely on the basis of being implanted with a BCI (Gilbert 2012; Gilbert 2013b; Gilbert et al. 2014).

BCIs offer great potential to enhance quality of life—in particular by increasing control over neurologic symptoms—but also involve challenges of potential unwanted outcomes that could make patients more vulnerable and subject to iatrogenic harms. This is supported by the results of our interviews with six patients, one of whom reported feelings of postoperative self-estrangement and a failure to achieve embodied fusion of self with the device. Evidence-based preparedness protocols specific to BCI technologies could prevent such potential harms. Physicians and patients would benefit from decision-making frameworks for relevant healthcare choices as developed from early ongoing experimental trials.

## Appendix: Semi-Structured Interview Script

These are examples of generic questions. They are an indication of the structure to be followed during the interviews, rather than the actual questions to be asked to patients. The choice of words, terminology or languages may change slightly for each patient.^6^

1. Potential questions regarding postoperative sense of the self.
  - What was it like to live without the predictive brain computer interface device (BCI) device? Did you feel that your medical condition affected who you were, your identity, or did you feel comfortable in yourself with your condition?
  - What is it like to be implanted with BCI? Do you feel any significant difference from before the device compared to after it was implanted?
    - If yes, please provide some examples. Did you expect to find differences or changes to yourself prior to the operation?
  - Would you agree with such statement: “the predictive BCI device has changed who I am”?
    - If yes, do you interpret this change as a form of enhancement or restoration of yourself? How?
    - If no, do you view the device as an integral part of who you are? Why?
  - Have others commented on any changes to your “self” (personality) since being implanted? If so, do you agree with them? Why or why not?
  - Do you think that you may change/change more in the future as a result of this intervention?
2. Potential questions regarding the sense of control.
  - Do you feel the device has increased your autonomy (making you less dependent on others)? For instance, has it given you back more control over your life? For example has it improved your life, control on symptoms, or sense of self?
  - Prior to the implantation of this device, how would you describe your control over your life?
  - If you experience the BCI device as s form of control, does this “control” feel authentic? Do you endorse it? From your own personal experience, do you see it as novel or restorative control?
  - Have others commented that you have better control over your life/symptoms/yourself? Do you agree with them? If not, why not?

## Footnotes

1 These references are not exhaustive. A large amount of work raises concerns or suggests the possibility of the impact of BCI on sense of identity, autonomy and self. Further work discussing these issues are Klein et al. (2015), Clausen (2011), Glannon and Ineichen (2016), Moritz et al. (2016), Trimper et al. (2014), Tamburrini (2009), Nijboer et al. (2013), Gilbert and Cook (2015), Clausen et al. (2017), Goering et al. (2017), Hongladarom (2015), McCullagh et al. (2014), Kotchetkov et al. (2010), Kyselo (2013), Hildt (2011), Abbott and Peck (2017), Jebari (2013), Vlek et al. (2012), Heersmink (2013), Lee (2016), Jebari and Hansson (2013), Racine et al. (2017).

2 This study was conducted in accordance with Tasmanian Human Research Ethics Committee regulations. Patient Consent and Minimal Risk Ethics Application Approval, entitled “(H0013883) Implantable Seizure Advisory Brain Devices: Ethical Implications” is in compliance with the Tasmanian Human Research Ethics Committee regulations. Initial ethics approval was obtained in March 2013, and an amendment was approved on November 2014.

3 Patient 3 self-reported past episodes of depression (prior to BCI implantation).

4 Postoperative self-estrangement is not necessarily a negative consequence. Capacities induced by clinical/medical/therapeutic/neurotechnological/DBS interventions are often experienced positively by patients as seen in the case of Patient 6. However, in some cases of patients implanted with open-loop DBS, postoperative feelings of estrangement largely appear to be correlated with a sense of drastic incapacity to re-identify with the previous self, which reflects an involuntary and unintentional shift in the experience of the self (Gilbert et al 2017).

5 Families and social context are an essential measurement of how patients experience potential estrangement, even if patients do not perceive it. Even if the patient doesn't report feelings of postoperative self-estrangement, their behaviour within their family and social context can show otherwise. An extended study of ours should not only involve interviewing the implanted patients, but also their close relatives.
